# Temporal trend and influencing factors of high-risk pregnancy in China: a nationwide longitudinal analysis

**DOI:** 10.3389/fpubh.2026.1818893

**Published:** 2026-06-29

**Authors:** Zhenyan Bo, Kun Zou, Hailong Li, Yuxin Jiang, Linan Zeng, Yong Tang, Shaoyang Zhao, Yongmu Jiang, Lingli Zhang

**Affiliations:** 1 Department of Pharmacy/Evidence-Based Pharmacy Center, West China Second University Hospital, Sichuan University, Chengdu, Sichuan, China; 2 Children's Medicine Key Laboratory of Sichuan Province, Chengdu, Sichuan, China; 3 NMPA Key Laboratory for Technical Research on Drug Products In Vitro and In Vivo Correlation, Chengdu, Sichuan, China; 4 Key Laboratory of Birth Defects and Related Diseases of Women and Children, Sichuan University, Ministry of Education, Chengdu, Sichuan, China; 5 Chinese Evidence-based Medicine Center, West China Hospital, Sichuan University, Chengdu, Sichuan, China; 6 School of Mathematics, Sichuan University, Chengdu, Sichuan, China; 7 West China Biomedical Big Data Center, West China Hospital, Sichuan University, Chengdu, Sichuan, China; 8 School of Economics, Sichuan University, Chengdu, Sichuan, China

**Keywords:** high-risk pregnancy, JoinPiont regression, linear mixed model, maternal health, maternal mortality rate

## Abstract

**Background:**

High-risk pregnancy was a major challenge to sustainably reduce maternal mortality rate to achieve the Sustainable Development Goals in China. This study aimed to analyze the temporal trend of high-risk pregnancy rate in China and to explore the influence of socioeconomic factors.

**Methods:**

Panel data of 31 provinces in China from 2002 to 2016 were extracted from the China Statistical Yearbooks and Health Statistical Yearbooks. We firstly adapted JoinPoint regression model to describe the temporal trend of high-risk pregnancy rate. Then, an auto-regressive integrated moving average model was built to predict high-risk pregnancy rate from 2017 to 2030. Finally, a linear mixed model was employed to evaluate the impact of socioeconomic factors on high-risk pregnancy rate at the provincial level.

**Results:**

The high-risk pregnancy rate increased substantially with an average annual percent change of 6.06% (95% CI: 5.70, 6.51%) from 1996 to 2016 in China. Except Tibet, the high-risk pregnancy rate of other 30 provinces all presented an upward trend with an average annual percent change ranging from 1.86% (95% CI: 1.47, 2.35%) to 11.15% (95% CI: 9.22, 13.10%). The predicted high-risk pregnancy rate would up to 38.76% (95% CI: 31.66, 45.85%) in 2030 in China. The number of hospital beds per 1,000 population was negatively associated with high-risk pregnancy rate (*β* = −0.0476, 95% CI: −0.0767, −0.0185). While female illiteracy and GRP per capita were positively correlated with high-risk pregnancy rate, the coefficient was 0.0127 (95% CI: 0.0050, 0.0203) and 0.1717 (95% CI: −0.0205, 0.3639) respectively.

**Conclusion:**

The high-risk pregnancy ratio in China has been increasing and sustains an upward trend, posing great challenge to maternal health improvement in China. Healthcare resources and other socioeconomic factors have significant effect on high-risk pregnant rate, such findings can provide evidence-based implications for long-term policy-making in healthcare resource allocation, urban development and gender equality. Constant efforts by all society should be made to enhance maternal health to achieve the goals of the Sustainable Development Goals and Healthy China 2030.

## 1 Introduction

High-risk pregnancy refers to a clinical classification of pregnancy in which the mother and/or the fetus (or newborn) is at increased risk of complications or adverse health outcomes. The consequences of high-risk pregnancy extend far beyond the immediate perinatal period, encompassing adverse maternal outcomes including increased morbidity and mortality (1), adverse fetal outcomes, such as intrauterine growth restriction and stillbirth (2), and adverse neonatal outcomes including preterm birth, low birth weight, and neonatal intensive care unit admission (3). International literature indicated that approximately 10–20% of pregnancies are classified as high-risk at the global level (2). In certain countries, this proportion reached considerably higher levels—for example, 20–30% in India (4), 26.4% in Ethiopia (5). An estimated 20 million women worldwide experienced high-risk pregnancy annually (4), which accounted for 70–80% of perinatal mortality and morbidity (6). In 2023, approximately 260,000 women died during and following pregnancy and childbirth, which meant that a maternal death occurred almost every 2 min according to the WHO. Notably, 92% of these deaths occurred in low- and lower-middle-income countries (7). Therefore, high-risk pregnancy has become a pressing public health priority requiring systematic investigation and targeted intervention.

Multiple factors can make a pregnancy high risk, with clinical risk factors at individual level serving as the most proximate determinants. These factors can be broadly categorized into maternal medical conditions, demographic characteristics, and behavioral attributes. Maternal health conditions constituted the major contributor. Pre-existing hypertension (8), diabetes mellitus (9), thyroid disorders (10), and cardiovascular disease (11) have all been consistently associated with increased pregnancy risk. A large population-based cohort study in Taiwan documented that the coexistence of gestational diabetes mellitus and hypertensive disorders significantly amplifies the risk of adverse maternal and neonatal outcomes (12). Maternal demographic factors also played a critical role. Advanced maternal age (≥35 years) has been identified as one of the most robust predictors of high-risk pregnancy (13). A retrospective cohort study concluded that women with maternal age over 40 years faced significantly higher risk of gestational diabetes, pregnancy-related hypertensive disorders and cesarean delivery compared with younger counterparts (14). Similarly, maternal stature (15), parity (16), and interpregnancy interval (17)all have been linked to pregnancy complications. Lifestyle and behavioral factors, including tobacco use, alcohol consumption (18), drug use (19) and inadequate prenatal care utilization (20), can further compound the risk profile. These clinical factors above provided essential insights for individual-level risk stratification and clinical management, however, they offered scant evidence for population-level interventions or policy guidance for high-risk pregnancy (21–23). A model that relied solely on patient-level clinical predictors cannot inform resource allocation decisions, health system planning, or equity-targeted strategies across regions (24–26).

Maternal health, as reflected in indicators such as maternal mortality ratio, serves not only as a key metric of population health and health system performance but also as a critical marker of gender equality and human development (24). The Sustainable Development Goals explicitly recognize maternal health as a fundamental component of SDG 3 (Good Health and Wellbeing) and SDG 5 (Gender Equality), with Target 3.1 aiming to reduce the global maternal mortality ratio to less than 70 per 100,000 live births by 2030 (27). Moreover, the United Nations Development Programme’s Gender Inequality Index incorporated maternal mortality as one of its core components, underscoring the inextricable link between maternal outcomes and broader dimensions of human development, including empowerment and economic participation (28). Achieving sustainable improvements in maternal health therefore necessitates moving beyond the clinic to examine how macro-level social, economic, and structural conditions shape pregnancy risk at the population level. In this regard, a growing body of studies explored the profound influence of social determinants of health (SDH) on high-risk pregnancy. SDH was defined by the World Health Organization as the conditions in which people are born, grow, work, live and age, and the wider forces that shape the conditions of daily life (29). In the conceptual framework, SDH was categorized into structural determinants and intermediate determinants. Structural determinants are situated at the distal level of the causal chain of health outcomes and represent the “fundamental causes.” They include the socioeconomic and political context (e.g., macroeconomic environment, governance systems) and the resulting mechanisms of social stratification (e.g., inequalities in income, education, gender, race/ethnicity). Intermediate determinants are positioned at the proximal level and serve as the “mediating pathways” through which structural determinants exert their effects on health outcomes. These include material conditions, the psychosocial environment, behavioral/biological factors, and the health system. A landmark global analysis published in The Lancet Global Health in 2024 reaffirmed that maternal health outcomes are products of multifactorial processes involving social, economic, and political determinants, with structural inequities and systemic discrimination frequently associated with poor maternal outcomes for vulnerable populations (24).

China presented a particularly compelling context for examining the social determinants of high-risk pregnancy. Over the past two decades, the country has undergone profound social transformations that have reshaped the epidemiological landscape of maternal health. Rapid economic growth has lifted hundreds of millions out of poverty, yet has simultaneously created substantial regional disparities in wealth and resource distribution (30). The pace of urbanization has been equally dramatic with the urban population share surging from 36.22% in 2002 to 67.89% in 2025 (31), fundamentally altering living conditions and healthcare access. Concurrently, China has experienced a significant demographic transition characterized by declining fertility rates, delayed marriage and childbearing, and an increasing proportion of births to women of advanced maternal age and multiparous pregnancy caused by fertility policy (32, 33). Meanwhile, high-risk pregnancy in China exhibited pronounced regional disparities. Overall, economically developed regions reported higher ratio of high-risk pregnancy than less developed areas (34–36). This apparent paradox likely reflected a combination of genuine risk elevation (due to delayed childbearing and lifestyle factors) and detection effects (where superior screening capacity identified more cases of high-risk pregnancy that would remain undetected in resource-constrained settings) (37, 38). Furthermore, the coexistence of declining maternal mortality and rising high-risk pregnancy further underscores the complexity of China’s maternal health transition (39, 40).

Against this backdrop of rapid social change and persistent regional inequities, there is an urgent need to systematically characterize the temporal evolution of high-risk pregnancy and identify the underlying social determinants driving its population-level patterns, so as to inform evidence-based maternal health policies tailored to diverse regional contexts in China. But as far as we currently know, although the existing research about high-risk pregnancy in China has made contributions, several critical gaps remained that limited the capacity for evidence-based macro-level intervention. First, the current studies just analyzed the high-risk pregnancy in some province in China, no study has conducted a nationwide (including 31 provinces) analysis of high-risk pregnancy trends. Second, the temporal dynamics of high-risk pregnancy—including trend turning points, annual percent changes, and future projections—remained poorly characterized, limiting the capacity for proactive policy planning. Third, the effect of socioeconomic and health system factors on high-risk pregnancy has not been sufficiently explored. Corresponding to the gaps, this study aimed to address this through three integrated objectives. The first was to characterize the temporal trends of high-risk pregnancy across China’s 31 mainland provinces using and analyze the regional difference. The second objective was to project future trajectories of high-risk pregnancy through 2030 to provide evidence for forward-looking policy planning. The third objective was to identify the socioeconomic and healthcare determinants of provincial-level variation in high-risk pregnancy. Drawing upon the SDH framework and existing evidence, we hypothesized that: (1) high-risk pregnancy would exhibit a significant upward trend with identifiable reginal difference; (2) economic development and healthcare resource availability would emerge as significant determinants for high-risk pregnancy.

## 2 Materials and methods

### 2.1 Theoretical framework and variable selection

Consistent with the SDH framework established by WHO, 8 variables were selected to examine their associations with high-risk pregnancy at the provincial level in China. The structural determinants encompassed urbanization rate, gross regional product (GRP) per capital, birth rate, child dependency ratio, and female illiteracy rate. Urbanization rate captured the proportion of the population residing in urban areas, reflecting the degree of economic development and infrastructure modernization. GRP per capita served as a proxy for provincial economic prosperity and living standards. The birth rate reflected population-level fertility pressure and the demand for maternity services. The child dependency ratio captured the broader demographic burden within a population. Higher child dependency ratios imply greater resource allocation demands on households and communities, potentially diverting attention and resources away from intensive perinatal care. Female illiteracy rate served as a proxy for female educational attainment and health literacy, which influenced prenatal care-seeking behavior and adherence to high-risk pregnancy management protocols. These structural indicators represented the macro-level conditions that shaped the resource environment within which pregnancies occurred. The intermediate determinants included hospital beds per 1,000 population, health staff per 1,000 population, and the ratio of health expenditure to general public budget expenditure (HE/GPBE). Hospital bed density was a well-established indicator of healthcare infrastructure capacity, directly reflecting the availability of inpatient resources for maternity care. Health staff density captured the human resource capacity for delivering skilled maternal health services, including prenatal screening, high-risk identification, and obstetric emergency management. HE/GPBE directly measured the government’s political will and strategic priority accorded to health in the process of fiscal resource allocation. This indicator was theoretically preferable to total health expenditure as a percentage of GDP, as it directly measured the commitment of local government resources to health relative to other competing public expenditures.

### 2.2 Variable definition and data resource

In this study, high-risk pregnancy rate (HRPR) was regarded as the dependent variable. According to the National Health Statistical Yearbooks, HRPR was defined as the ratio of the number of pregnancies classified as high risk to the total number of livebirths. High-risk pregnancy classification followed the national standard criteria established by the former Ministry of Health, encompassing maternal medical conditions (e.g., hypertension, diabetes, heart disease), obstetric complications (e.g., abnormal fetal position, multiple pregnancy, placental abnormalities), and demographic risk factors (e.g., advanced maternal age, short stature, history of adverse pregnancy outcomes) (41, 42). The annual data of HRPR at both national and provincial level was extracted from the National Health Statistical Yearbooks released issued by the National Health Commission of the People’s Republic of China. But the time span of HRPR at the two level was different, the national HRPR spanned the period from 1996 to 2016, while the provincial HRPR covered the period from 2002 to 2016.

The provincial explanatory variables in this study including birth rate, female illiteracy, urbanization rate, child dependency ratio, HE/GPBE and GRP per capita (adjusted for inflation based on the Consumer Price Index) were all collected from the China Statistical Yearbook released by the National Bureau of Statistics of the People’s Republic of China. Hospital beds per 1,000 population, health staff per 1,000 population were obtained from the National Health Statistical Yearbooks. Detailed information pertaining to each of the aforementioned variables could be found in the Supplementary Table S1.

### 2.3 Statistical analysis

In this study, we performed a four-stage analytical strategy. In the first stage, we conducted descriptive analysis to depict the HRPR in different regions in China. In accordance with the region division developed by the National Bureau of Statistics, the 31 provinces in China were divided into four regions: the eastern, central, western and northeastern region. The region divisions in China reflected fundamental structural disparities in economic development, industrial composition, natural resource endowments, and demographic profiles (43, 44). The provinces included in each region were presented in the Supplementary Table S2.

In the second stage, we established JoinPoint regression models to characterize the temporal trend of HRPR at both the national and provincial level. Given the skewed distribution characteristic of the HRPR, a log-linear model was adopted to fit the temporal trend. The annual percent change (APC) was estimated to evaluate the specific change trend for each segment, and the average annual percent change (AAPC) was estimated to evaluate the overall change trend during the entire duration. The optimal number and location of joinpoints were determined using the Monte Carlo permutation test (*n* = 4,500 permutations) with the Bayesian Information Criterion (BIC) for model selection. A maximum of 2 joinpoints was allowed based on the 15-year observation window. Statistical significance was assessed at the two-sided *α* = 0.05 level. When multiple comparisons were involved, the Bonferroni correction was applied to adjust the statistical significance level.

In the third stage, we built an auto-regressive integrated moving average (ARIMA) model based on national data of HRPR between 1996 and 2016 to predict HRPR from 2017 to 2030. The construction of the ARIMA model followed the Box-Jenkins four-step procedure (38, 45). Step 1 --Model identification: stationarity was assessed using the Augmented Dickey–Fuller (ADF) test. If the original series was non-stationary, first-order differencing was applied. Step 2—Parameter estimation: autocorrelation function (ACF) and partial autocorrelation function (PACF) plots were examined to determine the orders of autoregressive (*p*) and moving average (*q*) components. Step 3—Model diagnostics: the Ljung–Box *Q*-test was applied to test for the absence of autocorrelation (white noise) in the residual, and the Shapiro–Wilk test and the *Q*–*Q* plot were used to evaluate the normality of the residual distribution. Step 4—Forecasting: the optimal model was selected based on the Akaike information criterion (AIC), mean absolute percentage error and mean absolute error., and used to generate forecasts through 2030 with 95% confidence intervals.

In the fourth stage, we firstly summarized the between-province variation by year and the within-province change of HRPR and other provincial variables. Subsequently, given the hierarchical structure of the panel data employed in this study, where repeated annual observations (Level 1) were nested within provinces (Level 2), we applied a two-level linear mixed effect model to evaluate the impact of social determinants of health (SDH) on HRPR. Therefore, both within- and between-province components of variation in high-risk pregnancy rate could be distinguished and quantified. In addition, an autoregressive covariance structure was fitted for residual effects to account for serial correlation in HRPR across time within the same province. The intraclass correlation coefficient (ICC) was calculated to quantify the proportion of total variance in HRPR attributable to between-province differences, prior to the inclusion of predictor variables. A substantial ICC value would indicate that provincial-level clustering effects on HRPR could not be neglected, thereby justifying the adoption of a mixed-effects modeling approach over conventional ordinary least squares (OLS) regression.

In addition, we adopted a sequential model-building approach to examine the robustness of the estimated associations between SDH indicators and HRPR. First, an empty model (Model 1) with no predictors was fitted to assess the magnitude of between-province variation and to estimate the ICC. Second, structural determinants were added to form Model 2. Third, intermediate determinants were further included to construct the full model (Model 3). Year dummy variables were incorporated into model and 3 to account for secular trends. The logarithm transformation was applied to GRP per capita to mitigate right-skewness. The change in between and within provinces variance were examined to evaluate the explanatory contribution of the progressively added variable blocks. AIC was used to evaluate the fit of different models. Variance inflation factors (VIF) were calculated for all predictors to assess multicollinearity, with a VIF threshold of 5 used as the criterion for identifying problematic collinearity.

Results were expressed as estimation with corresponding 95% confidence intervals (CI). Statistical significance was set as *p* < 0.05 in this study. The Joinpoint regression model was built using the Joinpoint Regression Program version 4.9.1.0 (National Cancer Institute, Bethesda, USA). The analysis of ARIMA model and linear mixed effect model was performed using R 4.4.0. Maps were produced in ArcGIS 10.8.

## 3 Results

### 3.1 Descriptive analysis of HRPR in China and different regions

**Figure 1:**
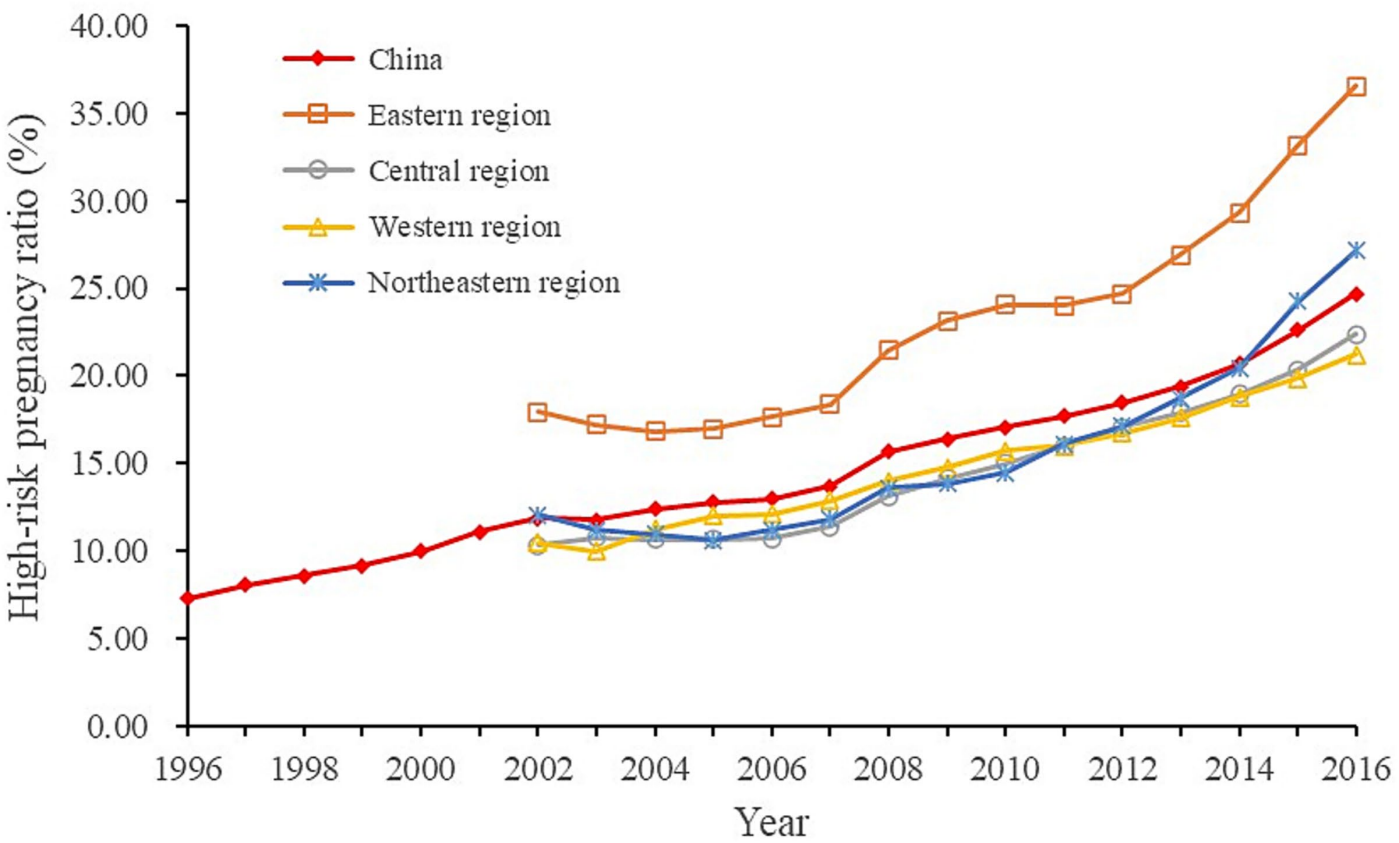
High-risk pregnancy rate in China from 1996 to 2016.

The national HRPR exhibited a notable increase from 7.30% in 1996 to 24.70% in 2016. The pattern of trend in HRPR was observed across all four regions, but the magnitude of increase varied. To illustrate, HRPR in eastern region drastically increased by 18.61% from 17.93% in 2002 to 36.54% in 2016. While the western region exhibited a 10.74% increase from 10.49% in 2002 to 21.23% in 2016. Moreover, in addition to the considerable variation in the magnitude of the increase in HRPR, there were significant discrepancies in the absolute HRPR between the eastern region and other regions. Notably, the HRPR in the eastern region was much higher than that observed in other three regions and the national level. Overall, the HRPR in the central, western and northeastern regions remained below the national level. However, it should be noted that the HRPR in the northeastern region began to exceed the national level from 2015 onwards (Figure 1).

### 3.2 Temporal trend and provincial variation of HRPR in China

**Figure 2:**
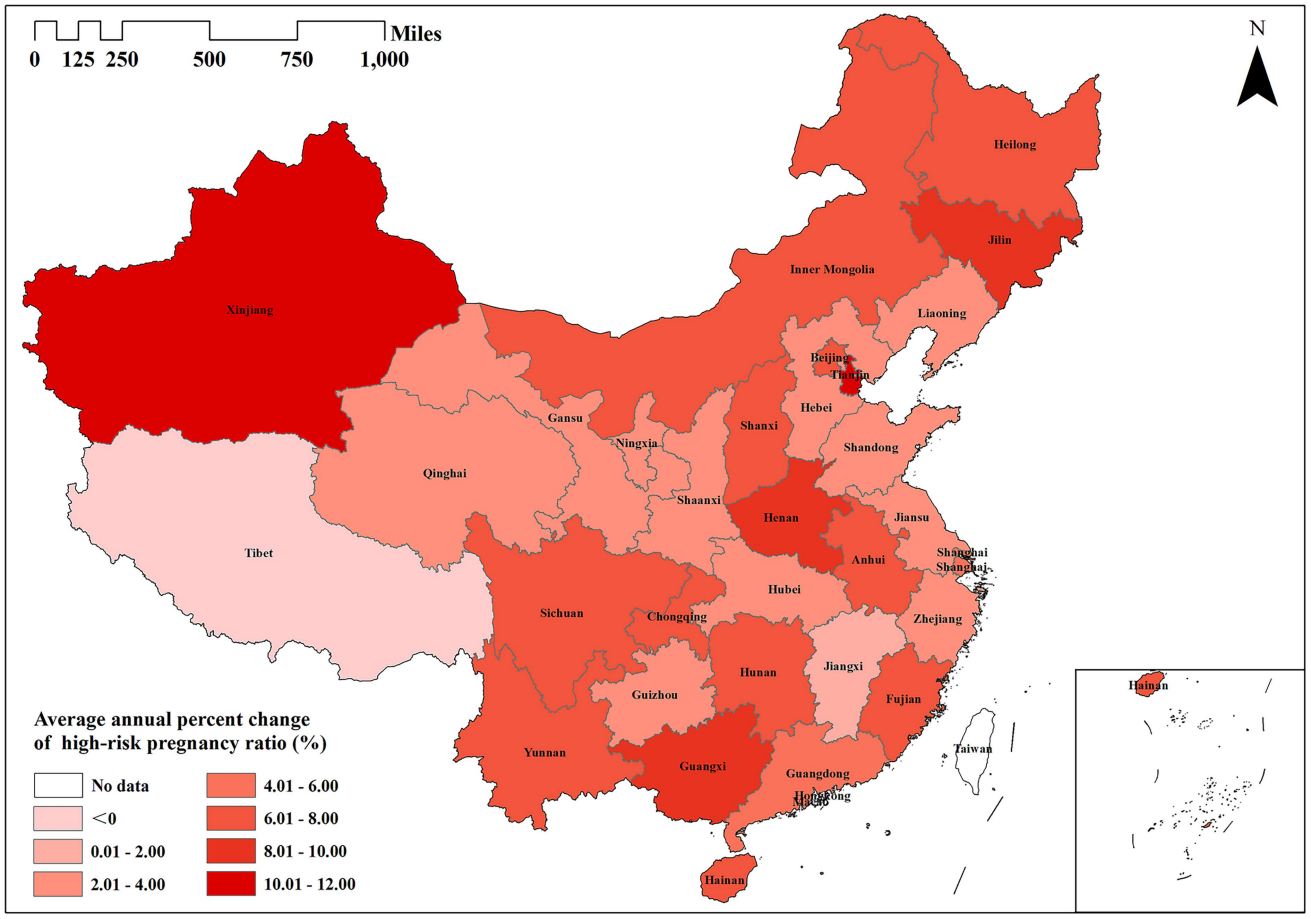
Average annual percent change of high-risk pregnancy rate by province in China from 2002 to 2016.

At the national level, the results of the Joinpoint regression model revealed that the HRPR encountered 2 distinct Joinpoints at 2002 and 2005, respectively. From1996 to 2002, the APC was 8.19% (95% CI: 6.84, 11.30%, *p* = 0.0004), from 2002 to 2005, the APC was 2.18% (95% CI: 0.57, 5.44%, *p* = 0.0164), from 2005 to 2016, the APC was 5.98% (95% CI: 5.11, 9.27%, *p* = 0.0044). The AAPC was 6.06% (95% CI: 5.70, 6.51%, *p* < 0.0001), indicating an overall upward trend of HRPR year by year from 1996 to 2016 in China. At the provincial level, only Tibet presented a declining trend, though this was not statistically significant (AAPC = −1.28, *p* = 0.2563). The HRPR of other 30 provinces all showed an increasing trend between 2002 and 2016 with the AAPC ranging from 1.86% (95% CI: 1.47, 2.35%) in Jiangxi province to 11.15% (95% CI: 9.22, 13.10%) in Xinjiang province (Figure 2). The APC for each province was shown in Supplementary Table S3.

### 3.3 Prediction of HRPR in China from 2017 to 2030

**Figure 3:**
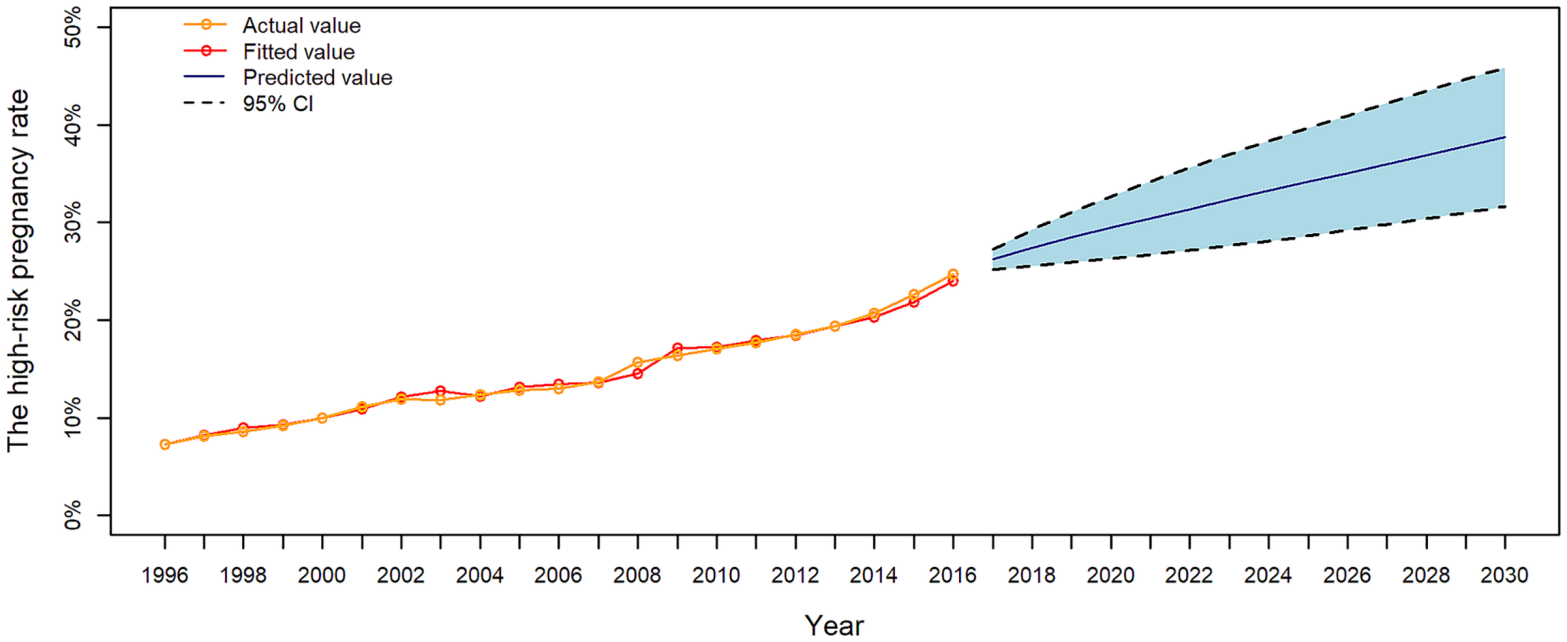
Predication of high-risk pregnancy rate in China from 2017 to 2030.

Due to data availability, the ARIMA model was built at the national level only. One difference transformation was used to ensure stationary of the time series of HRPR to the non-stationarity of the raw data. The final model was identified as ARIMA (1,1,0). The Akaike information criterion, correction, mean absolute error, and mean absolute percentage error of the model were 36.3314, 0.3522, and 2.4163% respectively, indicating a good model fit. The Ljung–Box *Q*-test demonstrated that all the residual sequence was white noise sequence (*χ*^2^ = 0.116, *p* = 0.7335), and both the visual inspection of the *Q*–*Q* plot (Supplementary Figure S1) and the Shapiro–Wilk test (*W* = 0.965, *p* = 0.628) supported the assumption of normally distributed residuals. The predicted values of HRPR from 2017 to 2023 were 26.20, 27.41, 28.48, 29.47, 30.43, 31.37, 32.30, 33.22, 34.15, 35.07, 35.99, 36.91, 37.83, 38.76%, respectively, (Figure 3). The detailed information of the predicted results was presented in the Supplementary Table S4.

### 3.4 Effect of socioeconomic factors on HRPR

Between-province variation and within-province changes of other socioeconomic factors and HRPR from 2002 to 2016 were reported in Table 1.

**Table 1:** Socioeconomic characteristics and high-risk pregnancy rate of 31 provinces in China.

Characteristics	Time variations (mean, 95% CI)			Within-province change, 2002 to 2016 (mean, 95% CI)
	2002	2008	2016	
Independent variables				
Hospital beds per 1,000 population	2.70 (2.38, 3.02)	3.36 (2.93, 3.79)	5.36 (5.11, 5.61)	2.66 (2.28, 3.03)
Birth rate, ‰	11.86 (10.52, 13.21)	11.42 (10.46, 12.38)	11.80 (10.72, 12.88)	−0.06 (−1.01, 0.89)
Female illiteracy, %	18.34 (14.74, 21.94)	12.91 (9.80, 16.02)	9.17 (6.11, 12.22)	−9.17 (−10.79, −7.56)
Urbanization rate, %	41.58 (35.69, 47.46)	48.32 (42.85, 53.79)	58.89 (54.36, 63.41)	17.31 (14.23, 20.39)
GRP per capita, 10,000 yuan	1.03 (0.74, 1.32)	2.61 (2.07, 3.16)	5.68 (4.73, 6.62)	4.64 (3.94, 5.35)
HE/GPBE	3.96 (3.68, 4.25)	5.56 (5.23, 5.89)	7.95 (7.39, 8.51)	−7.17 (−8.68, −5.67)
Child dependency ratio	30.14 (27.47, 32.80)	24.14 (21.54, 26.74)	22.96 (20.67, 25.26)	3.99 (3.38, 4.59)
Health staff per 1,000 population	3.89 (3.31, 4.47)	4.30 (3.57, 5.02)	6.23 (5.80, 6.65)	2.34 (1.95, 2.72)
Dependent variable				
High-risk pregnancy rate	13.01 (10.52, 15.50)	16.21 (13.45, 18.97)	26.97 (21.93, 32.02)	13.96 (10.51, 17.41)

GRP, gross reginal production; HE/GPBE, the ratio of health expenditure to general public budget expenditure.

Urbanization rate and health staff per 1,000 population were excluded from the preliminary regression model due to multicollinearity with GRP per capital (*r* = 0.9019) and hospital beds per 1,000 population (*r* = 0.8183) respectively, and the largest VIF value (VIF = 7.6711 for urbanization rate; VIF = 5.5658 for GRP per capital) (Supplementary Table S5). Results of all models were presented in Table 2. The empty model (model 1) yielded a considerable intraclass ICC of 0.6381, justifying the use of a multilevel modeling approach. Upon inclusion of structural determinants in Model 2, the within-province variance decreased substantially from 0.0904 to 0.0287, indicating that the model effectively captured the temporal fluctuations in HRPR within each province. Hospital beds per 1,000 population demonstrated a statistically significant negative association with the HRPR (*β* = −0.0639, 95% CI: −0.0952, −0.0325).

**Table 2:** The association of socioeconomical factors with high-risk pregnancy rate.

Variables	Log of high-risk pregnancy rate		
	Model 1	Model 2	Model 3
Fixed effect, *β* (95% CI)			
Hospital beds per 1,000 population	–	−0.0639^a^ (−0.0952, −0.0325)	−0.0476^a^ (−0.0767, −0.0185)
HE/GPBE	–	0.0183 (−0.0038, 0.0405)	0.0152 (−0.0075, 0.0380)
Birth rate, ‰	–	–	−0.0105 (−0.0297, 0.0087)
Female illiteracy, %	–	–	0.0127^a^ (0.0050, 0.0203)
Child dependency ratio	–	–	0.0016 (−0.0063, 0.0096)
Log of GRP per capital	–	–	0.1717^b^ (−0.0205, 0.3639)
Year	No	Yes	Yes
Random effects, variance (SE)			
Variance among provinces	0.1593 (0.0730)	0.1848 (0.0973)	0.1785 (0.9369)
Variance within provinces	0.0904 (0.0139)	0.0287 (0.0078)	0.0280 (0.0078)
Residual covariance			
ICC	0.6381	0.8657	0.8644
AIC	312.8135	−82.9119	−60.4204

^a,b^Denoted 5 and 10% significance level, respectively.

CI, confidence interval; ICC, intraclass correlation; AIC, Akaike information criterion; GRP, gross reginal production; HE/GPBE, the ratio of health expenditure to general public budget expenditure.

The full model (Model 3) further incorporated intermediate determinants. Hospital beds per 1,000 population continued to demonstrate significant associations with HRPR (*β* = −0.0476, 95% CI: −0.0767, −0.018), suggesting that each additional hospital bed per 1,000 population was associated with a significant decrease of approximately 6.38% of the HRPR. Female illiteracy rate showed a significant positive association with HRPR (*β* = 0.0127, 95% CI: 0.0050, 0.0203), implying that each one-percentage-point increase in female illiteracy was associated with an approximate 1.28% increase of HRPR. Birth rate, HE/GPBE and child dependency ratio showed no independent association effects. The effect of the logarithm of GRP per capita was positive but marginally non-significant (*β* = 0.1717, 95% CI: −0.0205, 0.3639), suggesting a possible positive link between economic development level and HRPR, though the statistical evidence remained inconclusive.

The AIC decreased substantially from 312.8135 in the null model to −60.4204 in model 3 which indicated a marked improvement in model fit. The rather higher ICC values for Models 2and 3 suggested persistent and substantial between-province heterogeneity that was not fully explained by the included predictors.

## 4 Discussion

Over the past three decades, China has made remarkable achievement in maternal health as a developing country with the world’s largest population (40, 46). Furthermore, the Chinese government has set an ambitious goal in the Health China 2030 to continuously reduce the maternal mortality rate to 12 per 100,000 livebirths (47). However, there are numerous new challenges to achieve the target, among which management of high-risk pregnancy has become one of the primary concerns (48). Aiming at this problem, this nationwide longitudinal analysis revealed three key findings, which were integrated within the SDH framework: (1) a significant sustained upward trend in HRPR across most provinces with pronounced regional disparities; (2) ARIMA projections suggested continued increases through 2030; and (3) hospital beds per 1,000 population, female illiteracy and GRP per capita were found to be significantly associated with HRPR, indicating that the continuous improvement of maternal health required the extensive mobilization of the whole society.

The upward trajectory of HRPR identified in this study was consistent with findings from previous researches. Wang and colleagues analyzed surveillance data from 31 provinces between 2008 and 2017 and reported that the median HRPR increased by 64.8%, from 14.87 to 24.50% (39). The magnitude and direction of high-risk pregnancy change aligned closely with the estimates in this study. A more recent study found that the prevalence of multiple coexisting risk factors (defined as two or more) rose from 25.8% in 2015 to 38.4% in 2020–2021 (34). At the provincial level, Chen et al. reported that the proportion of deliveries to women with advanced maternal age (≥35 years) in Hubei province increased from 14.6% during the one-child policy period to 31.6% during the universal two-child policy period (49), and Tian et al. documented a significant rise in pregnancy complications across 22 monitoring hospitals in Hebei province following the two-child policy adjustment in 2016 (32). Taken together, these studies confirmed that the increase in HRPR observed in this study reflected a genuine epidemiological shift rather than a measurement error.

The upward trend of HRPR in China might be mainly attributed to a few key direct factors. First, there has been a notable rise in disease burden caused by chronic non-communicable diseases in women over the past decades in China. Wen Peng and colleagues found that the prevalence of overweight/obesity, hypertension, diabetes and dyslipidemia among women increased significantly between 2002 and 2018. Specifically, the prevalence of overweight/obesity increased from 30.3 to 47.2%, hypertension from 18.0 to 24.2%, diabetes from 2.7 to 10.9%, and dyslipidemia from 15.9 to 27.1% (50). Second, the childbearing age was later than it used to be. A study showed that the mean age of first childbearing increased from 23.43 to 27.22 years old, and the average childbearing age increased from 26.12 to 28.98 years old between 1990 and 2020 in China (51). In addition, according to statistical data from the National Bureau of Statistics, the fertility rate among women aged 35–39 years group increased from 10.98 to 18.60% and between 2005 and 2015, while the rate increased from 2.05 to 5.37% among 40–44 years group during the same period (52). With the liberalization of fertility policies, the proportion of women with advanced maternal age might continue to rise at a higher level (53). Furthermore, assisted reproductive technology might be another contributing factor for the rising HRPR (54). At present, assisted reproductive technology has been a standard clinical practice to treat infertility. In 2016, the number of infants born through assisted reproductive technology accounted 1.69% of the total livebirths and showed an upward trend in China (55).

Up to now, the criteria of high-risk pregnancy classification has not been uniformed. Individual countries employed screening tools that varied widely in the number and type of risk factors included (3, 56). A systematic review and meta-analysis about multiple high-risk factors in pregnancy (MHFP) found a pooled global prevalence of 12% (95% CI: 12–13%), with substantial between-study heterogeneity driven by definitional differences: studies using more than 12 risk factors reported a MHFP prevalence of 15%, compared with 8% for those using only two factors (3). Notwithstanding these definitional differences, the upward trend in HRPR was not unique to China. The global meta-analysis documented an increasing trend in MHFP prevalence from 2001 to 2020 across both high-income and low- and middle-income settings (3). Meanwhile, the United States, the United Kingdom, and Australia have all reported concurrent increases in pregnancy complications (28). Studies indicated that some countries were experiencing a rebound in the maternal and child health due to the increase in high-risk pregnancy (57, 58). A recent study also predicted that the national infant mortality and under-five mortality would rise again since 2025 in China (59). The convergent upward trend in HRPR across diverse national contexts, irrespective of health system configuration, economic development level, or definition heterogeneity, indicated that rising high-risk pregnancy profiles have evolved from a country-specific concern into a global public health priority, which could compromise the progress toward Sustainable Development Goal targets for maternal mortality reduction.

The positive association between female illiteracy rate and HRPR observed in this study can be understood through three convergent pathways according to the SDH framework. The first pathway was the related health literacy and lifestyle risk accumulation. Individuals with low health literacy might be more likely to engage in behaviors that increase their risk of developing chronic non-communicable diseases, such as smoking, excessive alcohol consumption, and consuming a diet high in salt and sugar (60). Studies had showed that a lower education level was associated with higher incidence of diabetes, hypertension and stroke among women (61–63). The second pathway operated through the use of maternal health services. Research has demonstrated a strong correlation between low educational attainment and diminished income and socioeconomic status, which presented a significant barrier to accessing health services (64, 65). On one hand, women with low income and socioeconomic status have worse access to healthcare for timely diagnosis and treatment for chronic non-communicable diseases. On the other hand, women’s educational attainment was directly correlated with utilization of maternal health service, which played a crucial role in the prevention of maternal death (66). A substantial body of evidence confirmed a positive association between female educational attainment and the use of antenatal care (ANC), institutional delivery, and postnatal care services (67, 68), which increased the likelihood of unrecognized risk factors persisting into pregnancy. A longitudinal study analyzing maternal health service use and maternal mortality across Chinese provinces from 2009 to 2016 further underscored that education level and household income were crucial determinants of maternal health service access, with their protective effects being particularly pronounced in central and western regions (69). The third pathway concerned women’s capacity to access to healthcare resources within family and community structures. Illiterate women often face constrained autonomy in health-related decision-making, particularly in rural areas where patriarchal norms and resource control by male household heads still predominated. A multilevel analysis of demographic and health survey data from high-fertility sub-Saharan African countries found that women with no formal education had significantly lower odds of participating in healthcare decision-making compared with those with primary education (70). In China, although gender equality has advanced considerably in urban areas, traditional family structures in rural areas continue to limit women’s independent health-seeking behavior (71, 72). Studies among rural-to-urban migrant women in Shanghai identified low educational level as a key barrier to adequate ANC utilization, with many respondents reporting that they “did not know how many visits they should have” or “did not think they should have so many visits” (12, 73). In a word, the knowledge-attitude-behavior gap was compounded by limited autonomy in healthcare decision-making: women who cannot independently assess their health needs or use the healthcare system become dependent on family members’ willingness and ability to facilitate care access.

In this study, hospital beds per 1,000 population was proven to be a powerful protective factor for HRPR. For each one additional hospital bed per 1,000 population, there was an average decrease of 6.38% in HRPR. From the demand-side perspective, hospital bed density shaped healthcare accessibility through multiple dimensions that directly influence pregnancy outcomes. Geographic accessibility was the most direct impact path. Regions with higher bed density typically have more extensive healthcare facilities network, which could reduce the physical distance and travel time required for women to reach obstetric services (74, 75). In the Chinese context, where the three-tiered maternal and child health (MCH) network was established to cover county, township, and village levels, bed density serves as a structural indicator of how well this network is resourced at the grassroots level (76).

Economic accessibility was equally important. In regions with sparse hospital bed supply, the scarcity of healthcare resources often drove up out-of-pocket costs for maternity services, creating financial barriers that disproportionately affect women from low-income households (77, 78). The National Academies of Sciences, Engineering, and Medicine have identified lack of access to healthcare—including financial barriers, transportation limitations, and provider shortages—as a systems-level risk factor for poor pregnancy outcomes, which was independent of individual medical risk (79). In China, although the New Cooperative Medical Scheme and urban employee medical insurance have expanded coverage, the effective reimbursement rate for maternal services varies considerably across provinces, and women in low bed-density regions may still face substantial cost burdens that deter timely care-seeking (80).

Beyond mere availability, bed density is closely intertwined with the quality of obstetric care. In healthcare systems where bed expansion is accompanied by proportional investment in human resources, higher bed density correlates with better staffing ratios and more comprehensive service capabilities. A nationwide survey of ICU resources in China found that physician-to-bed ratios and nurse-to-bed ratios varied substantially across provinces, ranging from 0.39:1 to 0.75:1 for physicians and from 1.04:1 to 1.91:1 for nurses (81). Although this study focused on critical care rather than obstetrics, the underlying principle applies: bed density alone does not guarantee quality care if staffing levels are inadequate. In sub-Saharan Africa, research has demonstrated that every additional physician per 1,000 population increased the likelihood of urine testing during antenatal care by 19.8% and blood pressure monitoring by 1.1%, while nurse/midwife density was positively associated with a broad range of maternal health services including iron supplementation and HIV testing (82). These findings suggested that bed density, as a proxy for overall healthcare system resourcing, captures not only physical capacity but also the diagnostic and therapeutic capabilities essential for pregnancy risk management. However, this quantitative relationship between hospital beds density and HRPR warranted nuanced interpretation. The observed association likely reflected not merely the availability of hospital beds but also the broader capacity of the healthcare system to identify and manage pregnancy risks. Hospital bed density served as a proxy for institutional readiness, including the availability of trained obstetric personnel, diagnostic equipment, and referral pathways. Regions with higher hospital bed density might also have more developed systems for routine risk screening during pregnancy, enabling earlier identification and proactive management of complications. Conversely, in regions where hospital bed expansion has outpaced improvements in service quality or staffing, the marginal protective effect of additional beds might be attenuated.

The positive association between GDP per capita and HRPR can be understood through three convergent pathways of health transition during rapid economic development: the epidemiologic transition, the nutrition transition, and the demographic transition toward delayed childbearing. Omran’s theory of epidemiologic transition posits that as societies develop economically, the dominant pattern of disease shifts from infectious and communicable diseases toward chronic, degenerative conditions (83). This transition was not a passive consequence of economic growth but is driven by interconnected changes in living standards, sanitation, healthcare access, and lifestyle. In the context of maternal health, the critical implication was that the primary threats to pregnancy outcomes evolved: whereas infectious diseases and nutritional deficiencies historically dominated in low-income settings, chronic metabolic conditions—hypertension, diabetes, and obesity—have become the leading risk factors in rapidly developing economies (24, 84). Popkin’s concept of the nutrition transition provides a complementary mechanism, describing how economic development systematically alters dietary patterns and physical activity levels in ways that elevate obesity and diet-related non-communicable diseases (85, 86). The impact on pregnancy health was most directly observable in the rising prevalence of gestational diabetes mellitus (GDM). In urban China, the GDM rate climbed from 2.4% in 1999 to 8.1% in 2012—a nearly 3.5-fold increase (87). As GDM was both a direct high-risk classification criterion and a marker of underlying insulin resistance that predisposed to other pregnancy complications, the nutrition transition represented a major contributor to the observed positive GDP-HRPR association. The third pathway operated through the demographic consequences of economic development. Rising educational attainment, expanded career opportunities for women, and increased economic independence contributed to a systematic postponement of childbearing (88, 89). This trend, initially documented in high-income countries, has become increasingly prevalent in emerging economies. In the Chinese context, higher GDP per capita was strongly correlated with higher female educational attainment and greater participation in professional occupations (90). A nine-year retrospective cohort study at a tertiary hospital in China found that the trend in advanced maternal age increased by 75% between 2011 and 2019, when stratified by education and occupation, the increase was most pronounced among women with higher education levels and those in professional service occupations (89).

The HRPR was the highest in China’s most economically developed eastern region, while remaining lower in less-developed central, western, and northeastern regions. This “development paradox,” wherein maternal health risk indicators were elevated rather than attenuated in high GRP setting, parallelled with the effect of GRP per capital on HRPR. The SDH framework provided a structured lens for interpreting this paradox, in which structural determinants (institutional racism, discriminatory policies, historical disinvestment) shaped the distribution of social determinants (education, income, housing, healthcare access), which in turn drive maternal health inequities (91, 92). In the Chinese context, the structural determinant of unequal regional development policy—historically favoring coastal provinces through preferential economic zones and fiscal decentralization—has produced a skewed distribution of intermediary resources. Eastern provinces accumulated greater GDP per capita and urbanization capacity, but simultaneously experienced steeper epidemiologic and nutrition transitions, leading to higher chronic disease burden among women of reproductive age and greater proportions of advanced maternal age pregnancies.

Taken together, the “development paradox” documented in this study underscored a fundamental tension in maternal health policy: economic development generated both protective resources (healthcare infrastructure, education, insurance coverage) and risk-inducing transformations (chronic disease burden, dietary change, fertility delay). The net effect on HRPR depended on which pathway dominated, and in rapidly developing economies, the risk-inducing pathways might temporarily outpace the protective ones. Therefore, reducing maternal health inequities required not merely expanding healthcare resources, but also addressing the upstream structural and intermediary determinants—chronic disease prevention, nutritional education, fertility planning, and equitable resource allocation—that shaped the distribution of pregnancy risk across regions and populations.

There were some potential limitations in this study that should be acknowledged. First, the data of HRPR used in this study were extracted from the Health Statistics Yearbook, which was a guarantee for the data quality. However, high-risk pregnancy was screened using the comprehensive high-risk score method for pregnant women in the Health Statistics Yearbook prior to 2016, which was regarded as lacking in specificity and organization (93). Therefore, it was possible that the number of HRPR from 2017 to 2030 might be underestimated in this study. Second, the ARIMA model was deemed more appropriate for short-term prediction, the long-term trend prediction for 2025–2030 might be less accurate. However, we believed that the rising trend of HRPR was reliable and provided valuable insight into the status of maternal health in China. Third, aggregate provincial-level data precluded the examination of individual-level risk factors and may mask within-province heterogeneity. Future microdata research incorporating maternal age, parity, BMI, and medical history would enable more nuanced analyses. Fourth, the included independent variables, while theoretically grounded, did not exhaust potential HRPR determinants. Factors such as air pollution, occupational exposures, maternal mental health, and cultural practices were not included due to data constraints. Last, the linear mixed effect model captured associations rather than causal relationships. Potential endogeneity issues limit causal interpretation. We addressed these through Hausman testing, robust standard errors, and sensitivity analyses, but readers should interpret coefficients as exploratory associations. Despite the linear mixed effect model was grounded in the SDH framework and relevant covariates were included, residual endogeneity might remain due to unmeasured confounding, potential simultaneity, and measurement error. Thus, without access to exogenous variation or valid instrumental variables, our estimates should be interpreted as conditional associations rather than causal effects.

## 5 Conclusion

This nationwide longitudinal analysis revealed a significant sustained upward trend in HRPR across different provinces from 2002 to 2016 in China, with pronounced regional disparities characterized by a “development paradox.” The ARIMA model projected continued increases through 2030, though these long-term forecasts should be interpreted with caution. The linear mixed effect model identified that higher hospital beds per 1,000 population, along with lower female illiteracy and GRP per capital were associated with reduced HRPR. These findings illuminated the complex and paradoxical relationships between economic development, lifestyle transition, and healthcare system evolution in shaping maternal health outcomes. The rising burden of high-risk pregnancy in China reflected not merely clinical challenges but deeply rooted social and demographic transformations that demand differentiated, evidence-based policy responses. Specifically speaking, for developed eastern regions: Prioritizing chronic disease prevention programs targeting women of reproductive age, enhancing lifestyle intervention programs, and improving screening for gestational diabetes and hypertensive disorders. For developing western and central regions: Strengthening healthcare infrastructure (particularly hospital bed density and skilled birth attendance), expanding health insurance coverage for maternity services, and implement targeted health education programs. For northeastern regions: Addressing the emerging trend of HRPR exceeding national levels by focusing on the unique demographic challenges of population aging and outmigration. Nationwide policies: Investing in female education to enhance health literacy; establish dynamic healthcare resource allocation mechanisms responsive to changing pregnancy risk profiles; and strengthening the three-tier maternal and child health network. Future research should prioritize quasi-experimental designs utilizing policy shocks, individual-level longitudinal studies with comprehensive risk factor assessment, mechanism studies employing mediation analysis, and cross-national comparative analyses to advance understanding of maternal health determinants in transitioning economies.

## Data Availability

The datasets presented in this study can be found in online repositories. The names of the repository/repositories and accession number(s) can be found at: http://www.nhc.gov.cn/mohwsbwstjxxzx/tjtjnj/tjsj_list.shtml, https://www.stats.gov.cn/sj/ndsj/.

## References

[1] Crump C Sundquist J Sundquist K. Adverse pregnancy outcomes and long-term mortality in women. JAMA Intern Med. (2024) 184:631–40. doi: 10.1001/jamainternmed.2024.0276, 38619848 PMC11019441

[2] Sokou R Lianou A Lampridou M Panagiotounakou P Kafalidis G Paliatsiou S . Neonates at risk: understanding the impact of high-risk pregnancies on neonatal health. Medicina. (2025) 61:1077. doi: 10.3390/medicina61061077, 40572764 PMC12194930

[3] Zhang Y Ding W Wu T Wu S Wang H Fawad M . Pregnancy with multiple high-risk factors: a systematic review and meta-analysis. J Glob Health. (2025) 15:04027. doi: 10.7189/jogh.15.04027, 39913559 PMC11893144

[4] Johnson A Vaithilingan S Ragunathan L. Quantifying the occurrence of high-risk pregnancy: a comprehensive survey. Cureus. (2024) 16:e59040. doi: 10.7759/cureus.59040 38800298 PMC11128061

[5] Nesro J Kitila M Gelan M. Prevalence of high risk pregnant women who attend antenatal care and associated factors in Jimma medical center, Jimma town, South Western Ethiopia. Int J Women's Health Wellness. (2021) 31, 7:7. doi: 10.23937/2474-1353/1510133

[6] Chemir F Alemseged F Workneh D. Satisfaction with focused antenatal care service and associated factors among pregnant women attending focused antenatal care at health centers in Jimma town, Jimma zone, south West Ethiopia; a facility based cross-sectional study triangulated with qualitative study. BMC Res Notes. (2014) 7:164. doi: 10.1186/1756-0500-7-164 24646407 PMC3994781

[7] World Health Organization. Maternal mortality. (2025). Available online at: https://www.who.int/news-room/fact-sheets/detail/maternal-mortality (Accessed June 2, 2025).

[8] Perejón D Bardalet A Gascó I Siscart J Serna MC Orós M. Hypertension subtypes and adverse maternal and perinatal outcomes - a retrospective population-based cohort study. BMC Pregnancy Childbirth. (2024) 24:568. doi: 10.1186/s12884-024-06754-y, 39215229 PMC11363602

[9] Mahmoud E Elsayed AM Elsayed B Elsalakawi Y Gopinath A Chivese T. Association between gestational diabetes mellitus diagnostic criteria and adverse pregnancy outcomes—a systematic review and meta-analysis of adjusted effect sizes from studies using current diagnostic criteria. BMJ Open. (2024) 14:e091258. doi: 10.1136/bmjopen-2024-091258, 39578035 PMC11590801

[10] Puthiyachirakal MA Hopkins M AlNatsheh T Das A. Ountverview of thyroid disorders in pregnancy. Maternal Health Neonatol Perinatol. (2025) 11:9. doi: 10.1186/s40748-025-00208-9, 40170119 PMC11963318

[11] Türkmen O Akar İnan S. Navigating pregnancy with cardiovascular disease: pathophysiology, risk stratification, and maternal–fetal outcomes. Turk J Med Sci. (2024) 55:24–42. doi: 10.55730/1300-0144.5940, 40104301 PMC11913499

[12] Venkatesh KK Grobman WA Wu J Shah NS Pencina M Costantine MM . Hypertensive disorders of pregnancy and gestational diabetes mellitus and predicted risk of maternal cardiovascular disease 10–14 years after delivery: a prospective cohort. Diabet Med. (2025) 42:e15516. doi: 10.1111/dme.15516, 39825470 PMC12005981

[13] Gill SK Broussard C Devine O Green RF Rasmussen SA Reefhuis J . Association between maternal age and birth defects of unknown etiology ― United States, 1997–2007. Birth Defects Res A Clin Mol Teratol. (2012) 94:1010–8. doi: 10.1002/bdra.23049, 22821755 PMC4532312

[14] Londero AP Rossetti E Pittini C Cagnacci A Driul L. Maternal age and the risk of adverse pregnancy outcomes: a retrospective cohort study. BMC Pregnancy Childbirth. (2019) 19:261. doi: 10.1186/s12884-019-2400-x, 31337350 PMC6651936

[15] Helmy E Lesimbang HB Hossain Parash MT Ruey S Kamarudin NB Siong OT . The association between maternal short stature and neonatal intensive care unit admission: a longitudinal study in Sabah. Cureus. (2023) 15:e48924. doi: 10.7759/cureus.48924 38106728 PMC10725517

[16] Liu Y Li B Liu J Wen B Xiao C Chen Y . Neonatal and maternal adverse outcomes among low-risk nulliparous women compared with multiparous women at 37–41 weeks of gestation: a cohort study in South China. Front Med. (2026) 12:2025. doi: 10.3389/fmed.2025.1691707, 41561938 PMC12813074

[17] Wen X Liang W Zhai J Wang Y Zheng P Wang S. The association between interpregnancy intervals and preterm birth: a systematic review and meta-analysis. BMC Pregnancy Childbirth. (2025) 25:226. doi: 10.1186/s12884-025-07259-y, 40022000 PMC11871605

[18] Cooper KM Patel AK Kaluri S Devuni D. Pregnancy and liver-related outcomes after alcohol-associated hepatitis: a global multicenter study. Hepatol Commun. (2025) 9:e0663. doi: 10.1097/hc9.0000000000000663, 40048443 PMC11888976

[19] Kandhasamy S Lepigeon K Baggio S Céline R Ceulemans M Winterfeld U . Risk of adverse obstetrical and neonatal outcomes in women consuming recreational drugs during pregnancy. BMC Pregnancy Childbirth. (2025) 25:456. doi: 10.1186/s12884-024-07062-1, 40240903 PMC12004786

[20] Wallace ME Vilda D Dyer L Johnson I Funke L. Health care use and health consequences of geographic lack of access to abortion and maternity care. Birth (Berkeley, Calif). (2024) 51:363–72. doi: 10.1111/birt.12792, 37968858 PMC11093883

[21] Castagna C Huff A Douglas A Garofano M Fabi M Hass R . Stratifying the population based on health risk: identification of patient key health risk factors through consensus techniques. BMC Primary Care. (2025) 26:229. doi: 10.1186/s12875-025-02923-w, 40684090 PMC12275309

[22] Utomo B Romadlona NA Naviandi U BaharuddinNur RJ Makalew R Liyanto E . Census block based loglinear regression analysis of health and social determinants of maternal mortality in Indonesia 2010-2021. Sci Rep. (2025) 15:9397. doi: 10.1038/s41598-025-91942-9, 40102491 PMC11920268

[23] Vousden N Geddes-Barton D Hanley SJ Roberts N Knight M. Interventions to reduce inequalities for pregnant women living with disadvantage in high-income countries: an umbrella review. BMC Public Health. (2025) 25:1140. doi: 10.1186/s12889-025-22283-5, 40133955 PMC11938774

[24] Souza JP Day LT Rezende-Gomes AC Zhang J Mori R Baguiya A . A global analysis of the determinants of maternal health and transitions in maternal mortality. Lancet Glob Health. (2024) 12:e306–16. doi: 10.1016/S2214-109X(23)00468-0, 38070536

[25] Crowley C Harner B Stuck AR Kent T. New healthcare payment models: risk scores aren’t enough to guide resource allocation. Sci Rep. (2025) 15:18917. doi: 10.1038/s41598-025-04285-w 40442243 PMC12122729

[26] Galea S Keyes KM. Population health science and the challenges of prediction. Ann Intern Med. (2017) 167:511–2. doi: 10.7326/m17-1733, 28847011 PMC5931711

[27] The United Naitons. Goal 3: good health and well-being. SDG targets and indicators. (2026). Available online at: https://data.unicef.org/sdgs/goal-3-good-health-wellbeing/ (Accessed June 2, 2026).

[28] United Nations Development Programme. Gender Inequality Index (GII). Human Development Reports. (2026). Available online at: https://hdr.undp.org/data-center/thematic-composite-indices/gender-inequality-index#/indicies/GII (Accessed June 2, 2026).

[29] World Health Organization. Social Determinants of Health. (2015). Available online at: https://www.who.int/news-room/fact-sheets/detail/social-determinants-of-health (Accessed June 2, 2026).

[30] Xiang X He S Abid A. Toward SDG 3 in a developing nation: the role of health expenditure, financial development, and population growth in shaping health outcomes. Front Public Health. (2025) 13:1657624. doi: 10.3389/fpubh.2025.1657624, 40969627 PMC12440870

[31] National Bureau of Statistics of China. China Statistical Yearbook. (2016). Available online at: https://data.stats.gov.cn/dg/website/page.html#/pc/national/en/home (Accessed June 2, 2016).

[32] Tian M-L Ma G-J Du L-Y Jin Y Zhang C Xiao Y-G . The effect of 2016 Chinese second-child policy and different maternal age on pregnancy outcomes in Hebei Province, China. BMC Pregnancy Childbirth. (2023) 23:267. doi: 10.1186/s12884-023-05552-2, 37076792 PMC10114327

[33] Li HT Xue M Hellerstein S Cai Y Gao Y Zhang Y . Association of China's universal two child policy with changes in births and birth related health factors: national, descriptive comparative study. BMJ (Clin Res ed). (2019) 366:l4680. doi: 10.1136/bmj.l4680, 31434652 PMC6699592

[34] Zhang Y Ding W Dai X Wang H Cheng Y Dai J . Burden of multiple high-risk factors in pregnancy before and after the universal two-child policy in Chinese women: an observational study. J Glob Health. (2024) 14:04134. doi: 10.7189/jogh.14.04134, 39024620 PMC11257705

[35] Liu Y Luo R Huang A. The distribution of pregnant women with different pregnancy risks—4 cities, China, 2019. China CDC Week. (2021) 3:50–3. doi: 10.46234/ccdcw2021.016, 34594955 PMC8392934

[36] Yang X Wan Nordin N Ning Y Abdullah B Naing NN Chen XW. High-risk pregnancies according to five risk assessment tools in northwestern China: detection and parameters for diagnostic accuracy. Clin Epidemiol Glob Health. (2025) 36:102191. doi: 10.1016/j.cegh.2025.102191

[37] Ali ST Rizvi SA Talat M Abuzar S Azhar M Rehman M. Barriers to timely and adequate antenatal care: a systematic review of socioeconomic, cultural, psychosocial, and health-system factors across high and low resource settings. BMC Pregnancy Childbirth. (2025) 26:96. doi: 10.1186/s12884-025-08565-1, 41454267 PMC12849346

[38] Box GEP Jenkins GM. Time series analysis: forecasting and control. J Time Ser Anal. (2010) 31:303. doi: 10.1111/j.1467-9892.2009.00643.x

[39] Liu J Jing W Liu M. Risk management of pregnant women and the associated low maternal mortality from 2008-2017 in China: a national longitude study. BMC Health Serv Res. (2022) 22:335. doi: 10.1186/s12913-022-07721-z, 35287680 PMC8920427

[40] Qiao J Wang Y Li X Jiang F Zhang Y Ma J . A lancet commission on 70 years of women's reproductive, maternal, newborn, child, and adolescent health in China. Lancet (London, England). (2021) 397:2497–536. doi: 10.1016/s0140-6736(20)32708-2, 34043953

[41] National Health Commission of the People's Republic of China. The National Health Statistical Yearbooks. Available online at: https://www.nhc.gov.cn/mohwsbwstjxxzx/tjtjnj/tjsj_list.shtml (Accessed February 25, 2026).

[42] Zhu J Said FM Tan CH. Progress in research on the Management of High-Risk Pregnancies in China. Int J Biotechnol Biomed (IJBB). (2024) 1:44–56. doi: 10.31674/ijbb.2024.v01i02.005

[43] Zhao Z Cai Y. Research on the spatial differences and network structure of economic development in the Yangtze River belt, China. Sustainability. (2024) 16:5023. doi: 10.3390/su16125023

[44] Fan R Nie C Zhao Y Hao C Peng C. Spatiotemporal distribution and regional imbalance of China’s digital economy. Sustainability. (2024) 16:6738. doi: 10.3390/su16166738, 30654563

[45] Ljung GM Box GEP. On a measure of lack of fit in time series models. Biometrika. (1978) 65:297–303. doi: 10.2307/2335207

[46] Liang J Li X Kang C Wang Y Kulikoff XR Coates MM . Maternal mortality ratios in 2852 Chinese counties, 1996-2015, and achievement of millennium development goal 5 in China: a subnational analysis of the global burden of disease study 2016. Lancet (London, England). (2019) 393:241–52. doi: 10.1016/s0140-6736(18)31712-4, 30554785 PMC6336935

[47] The State Council of the People's Republic of China. The Healthy China 2030. (2016). Available online at: https://www.gov.cn/zhengce/2016-10/25/content_5124174.htm (Accessed February 25, 2026).

[48] Liu J Song L Qiu J Jing W Wang L Dai Y . Reducing maternal mortality in China in the era of the two-child policy. BMJ Glob Health. (2020) 5:e002157. doi: 10.1136/bmjgh-2019-002157, 32133196 PMC7042574

[49] Zhu C Zhang S Shen L Ye L Zhan M Cai S . Changes in the characteristics and outcomes of high-risk pregnant women who delivered prior to and after China's universal two-child policy: a real-world retrospective study, 2010-2021. BMC Public Health. (2024) 24:336. doi: 10.1186/s12889-024-17810-9, 38297279 PMC10829306

[50] Peng W Chen S Chen X Ma Y Wang T Sun X . Trends in major non-communicable diseases and related risk factors in China 2002-2019: an analysis of nationally representative survey data. Lancet Regional Health Western Pacific. (2024) 43:100809. doi: 10.1016/j.lanwpc.2023.100809, 38456095 PMC10920046

[51] Xianling Z Yinan S. Postponement of marriage and childbearing in China during 1990-2020: trend and characteristics. (in Chinese). Popul Res. (2023) 47:88–101.

[52] National Bureau of Statistics of China. China Statistical Yearbook 2016. Beijing: China Statistics Press (2017).

[53] Li H Nawsherwan Fan C Yin S Haq IU Mubarik S . Changes in adverse pregnancy outcomes in women with advanced maternal age (AMA) after the enactment of China's universal two-child policy. Sci Rep. (2022) 12:5048. doi: 10.1038/s41598-022-08396-6, 35322808 PMC8943149

[54] Tai W Hu L Wen J. Maternal and neonatal outcomes after assisted reproductive technology: a retrospective cohort study in China. Front Med. (2022) 9:837762. doi: 10.3389/fmed.2022.837762, 35479950 PMC9037083

[55] Bai F Wang DY Fan YJ Qiu J Wang L Dai Y . Assisted reproductive technology service availability, efficacy and safety in mainland China: 2016. Hum Reprod. (2020) 35:446–52. doi: 10.1093/humrep/dez245, 32020190

[56] Yang X Chen XW PRA B. Defining high-risk pregnancy: protocol for a systematic scoping review of clinical determinants, complications, and adverse birth outcomes. PLoS One. (2025) 20:e0334326. doi: 10.1371/journal.pone.0334326, 41091776 PMC12527127

[57] Carroll AE. Why is US maternal mortality rising? JAMA. (2017) 318:321. doi: 10.1001/jama.2017.8390, 28742896

[58] Harpur A Minton J Ramsay J McCartney G Fenton L Campbell H . Trends in infant mortality and stillbirth rates in Scotland by socio-economic position, 2000-2018: a longitudinal ecological study. BMC Public Health. (2021) 21:995. doi: 10.1186/s12889-021-10928-0, 34044796 PMC8155799

[59] Zhang M Qu H Xia J Hui X Shi C Xu F . Trends, influencing factors and prediction analysis of under-five and maternal mortality rates in China from 1991 to 2020. Front Public Health. (2023) 11:1198356. doi: 10.3389/fpubh.2023.1198356, 37927855 PMC10620530

[60] Taggart J Williams A Dennis S Newall A Shortus T Zwar N . A systematic review of interventions in primary care to improve health literacy for chronic disease behavioral risk factors. BMC Fam Pract. (2012) 13:49. doi: 10.1186/1471-2296-13-49, 22656188 PMC3444864

[61] Oshio T Kan M. Educational level as a predictor of the incidences of non-communicable diseases among middle-aged Japanese: a hazards-model analysis. BMC Public Health. (2019) 19:852. doi: 10.1186/s12889-019-7182-6, 31262277 PMC6604183

[62] Li J Ni J Wu Y Zhang H Liu J Tu J . Sex differences in the prevalence, awareness, treatment, and control of diabetes mellitus among adults aged 45 years and older in rural areas of northern China: a cross-sectional. Popul-Based Study Front Endocrinol. (2019) 10:147. doi: 10.3389/fendo.2019.00147, 30923514 PMC6426742

[63] Bin T Xin J Siqi Z Meng F Yang GU Songli R . Effects of education level on hypertension, diabetes, and dyslipidemia among residents in Hezhang County. Prev Med Rep. (2026) 62:103369. doi: 10.1016/j.pmedr.2025.103369, 41684864 PMC12891900

[64] The Lancet Public H. Education: a neglected social determinant of health. Lancet Public Health. (2020) 5:e361. doi: 10.1016/s2468-2667(20)30144-4, 32619534 PMC7326385

[65] Santos EFS Louvison MCP Oliveira ECT Monteiro CN Barros MBA Goldbaum M . Analysis of education level in access and use of health care services, ISA-Capital, São Paulo, Brazil, 2003 and 2015. Cad Saude Publica. (2023) 39:e00249122. doi: 10.1590/0102-311xen249122, 37820229 PMC10566551

[66] Wang H Frasco E Takesue R Tang K. Maternal education level and maternal healthcare utilization in the Democratic Republic of the Congo: an analysis of the multiple indicator cluster survey 2017/18. BMC Health Serv Res. (2021) 21:850. doi: 10.1186/s12913-021-06854-x, 34419033 PMC8380349

[67] Yang Y Yu M. Disparities and determinants of maternal health services utilization among women in poverty-stricken rural areas of China: a cross-sectional study. BMC Pregnancy Childbirth. (2023) 23:115. doi: 10.1186/s12884-023-05434-7, 36788495 PMC9926695

[68] Wu Y Zhou H Wang Q Cao M Medina A Rozelle S. Use of maternal health services among women in the ethnic rural areas of western China. BMC Health Serv Res. (2019) 19:179. doi: 10.1186/s12913-019-3996-2, 30890133 PMC6425603

[69] Luo M Ding H Chen X Luo P Dong E Li S. Socioeconomic and health system determinants of maternal health inequities across Chinese provinces (2009–2021): a multivariate meta-regression analysis of regional heterogeneity in underlying mechanisms. Front Public Health. (2026) 14:1722508. doi: 10.3389/fpubh.2026.1722508, 41783712 PMC12953505

[70] Negash W Kefale G Belachew T Asmamaw D. Married women decision making autonomy on health care utilization in high fertility sub-Saharan African countries: a multilevel analysis of recent demographic and health survey. PLoS One. (2023) 18:e0288603. doi: 10.1371/journal.pone.0288603, 37440579 PMC10343071

[71] Li J. Gender inequality, family planning, and maternal and child care in a rural Chinese county. Soc Sci Med. (2004) 59:695–708. doi: 10.1016/j.socscimed.2003.11.041 15177828

[72] Jia J Jia C Zhang X Ren P Chen M Xu J. The impact of internet medical service on rural gender inequality in health opportunity: a cross-sectional study. BMC Public Health. (2024) 24:3093. doi: 10.1186/s12889-024-20575-w, 39516739 PMC11545802

[73] Zhao Q Huang ZJ Yang S Pan J Smith B Xu B. The utilization of antenatal care among rural-to-urban migrant women in Shanghai:a hospital-based cross-sectional study. BMC Public Health. (2012) 12:1012. doi: 10.1186/1471-2458-12-1012, 23170773 PMC3577466

[74] Wong KLM Benova L Campbell OMR. A look back on how far to walk: systematic review and meta-analysis of physical access to skilled care for childbirth in sub-Saharan Africa. PLoS One. (2017) 12:e0184432. doi: 10.1371/journal.pone.0184432, 28910302 PMC5598961

[75] Hanson C Cox J Mbaruku G Manzi F Gabrysch S Schellenberg D . Maternal mortality and distance to facility-based obstetric care in rural southern Tanzania: a secondary analysis of cross-sectional census data in 226 000 households. Lancet Glob Health. (2015) 3:e387–95. doi: 10.1016/S2214-109X(15)00048-0 26004775

[76] Feng XL Martinez-Alvarez M Zhong J Xu J Yuan B Meng Q . Extending access to essential services against constraints: the three-tier health service delivery system in rural China (1949-1980). Int J Equity Health. (2017) 16:49. doi: 10.1186/s12939-017-0541-y, 28532500 PMC5441056

[77] Ahmed S Creanga AA Gillespie DG Tsui AO. Economic status, education and empowerment: implications for maternal health service utilization in developing countries. PLoS One. (2010) 5:e11190. doi: 10.1371/journal.pone.0011190, 20585646 PMC2890410

[78] Perkins M Brazier E Themmen E Bassane B Diallo D Mutunga A . Out-of-pocket costs for facility-based maternity care in three African countries. Health Policy Plan. (2009) 24:289–300. doi: 10.1093/heapol/czp013, 19346273 PMC2699243

[79] National Academies of Sciences E, and Medicine; Health and Medicine Division; Division of Behavioral and Social Sciences and Education; Board on Children, Youth, and Families; Committee on Assessing Health Outcomes by Birth Settings. In: Backes EP Scrimshaw SC, editors. Birth Settings in America: Outcomes, Quality, Access, and Choice. Cambridge: Academies Press (2020) 32049472

[80] Zhang H Shi L Yang J Sun G. Efficiency and equity of bed utilization in China’s health institutions: based on the rank-sum ratio method. Int J Equity Health. (2023) 22:177. doi: 10.1186/s12939-023-01986-4, 37660026 PMC10474721

[81] Zhang X Chen Y Liu R Fang W Qin B Jiang R . Critical care resource disparities in China: a nationwide survey and policy recommendations for health equity. Crit Care. (2026) 30:209. doi: 10.1186/s13054-026-05983-1, 41968326 PMC13122895

[82] Rosser JI Aluri KZ Kempinsky A Richardson S Bendavid E. The effect of healthcare worker density on maternal health service utilization in sub-Saharan Africa. Am J Trop Med Hygiene. (2022) 106:939–44. doi: 10.4269/ajtmh.21-0727, 35026729 PMC8922518

[83] Omran AR. The epidemiologic transition: a theory of the epidemiology of population change. Milbank Mem Fund Q. (1971) 49:509–38. doi: 10.2307/3349375, 5155251

[84] Say L Chou D Gemmill A Tunçalp Ö Moller A-B Daniels J . Global causes of maternal death: a WHO systematic analysis. Lancet Glob Health. (2014) 2:e323–33. doi: 10.1016/S2214-109X(14)70227-X, 25103301

[85] Popkin BM. The nutrition transition and obesity in the developing world. J Nutr. (2001) 131:871s–3s. doi: 10.1093/jn/131.3.871S, 11238777

[86] Popkin BM. Global nutrition dynamics: the world is shifting rapidly toward a diet linked with noncommunicable diseases2. Am J Clin Nutr. (2006) 84:289–98. doi: 10.1093/ajcn/84.1.289, 16895874

[87] Emanuel AL Nieuwenhoff MD Klaassen ES Verma A Kramer MHH Strijers R . Relationships between type 2 diabetes, neuropathy, and microvascular dysfunction: evidence from patients with cryptogenic axonal polyneuropathy. Diabetes Care. (2017) 40:583–90. doi: 10.2337/dc16-1690, 28202549

[88] Ding X Li H Yang Q Nawsherwan. The trend in delayed childbearing age and its potential impact on adverse maternal-perinatal outcomes in developed and developing countries: a narrative review. Iran J Public Health. (2025) 54:1–12. doi: 10.18502/ijph.v54i1.17570, 39902369 PMC11787829

[89] Li H Nawsherwan FC Mubarik S Nabi G Ping YX. The trend in delayed childbearing and its potential consequences on pregnancy outcomes: a single center 9-years retrospective cohort study in Hubei, China. BMC Pregnancy Childbirth. (2022) 22:514. doi: 10.1186/s12884-022-04807-8, 35751047 PMC9233367

[90] Wu X Ali A Zhang T Chen J Hu W. An empirical analysis of the impact of gender inequality and sex ratios at birth on China's economic growth. Front Psychol. (2022) 13:1003467. doi: 10.3389/fpsyg.2022.1003467, 36389497 PMC9645222

[91] Crear-Perry J Correa-de-Araujo R Lewis Johnson T McLemore MR Neilson E Wallace M. Social and structural determinants of health inequities in maternal health. J Women's Health. (2021) 30:230–5. doi: 10.1089/jwh.2020.8882, 33181043 PMC8020519

[92] World Health Organization. A Conceptual Framework for Action on the Social Determinants of Health. Geneva: WHO (2010).

[93] Zhang S Huang T. Value of improved risk early warning and evaluation management on improving perinatal outcome of high-risk pregnant women. (in Chinese). Chin Med Rec. (2019) 20:91–4.

