# Supplementary material for: Temporal trend and influencing factors of high-risk pregnancy in China: a nationwide longitudinal analysis
Source: Front Public Health. 2026 Jun 29;14:1818893. doi: 10.3389/fpubh.2026.1818893 (PMC13357421; doi:10.3389/fpubh.2026.1818893)
Supplement: Supplementary file 1 [file Data_Sheet_1.docx]

**Table S1 Description of included variables**

| **Variable** | **Resource** | **Time periods** | **Measurement** | **Units** | **Sampling techniques** |
| --- | --- | --- | --- | --- | --- |
| High-risk pregnancy ratio | National Health Statistical Yearbooks | 1996-2016 | Ratio of annual number of high-risk pregnant women to all live births | Per hundred | The surveillance data were collected from 116 randomly selected monitoring sites (328 in total between 1996 and 2006, 336 in total between 2007 and 2016) in 31 provinces. |
| Hospital beds per 1,000 population | National Health Statistical Yearbooks | 2002-2016 | Annual number of hospital beds (including all healthcare institutions except for clinics) per 1,000 population for 31 provinces. | Beds per 1,000 population | Healthcare institutions or health administrative institutions were required to regularly report this variable by a national reporting system to National Health Commission in China. |
| Health staff per 1000 population | National Health Statistical Yearbooks | 2002-2016 | Annual number of health staff per 1,000 population for 31 provinces. | Health staff per 1,000 population | Healthcare institutions or health administrative institutions were required to regularly report this variable by a national reporting system to National Health Commission in China. |
| HE/GPBE | National Health Statistical Yearbooks | 2002-2016 | Ratio of health expenditure to general public budget expenditure. | Per hundred | Healthcare institutions or health administrative institutions were required to regularly report this variable by a national reporting system to National Health Commission in China. |
| Birth rate | China Statistical Yearbook | 2002-2004, 2006-2009, 2011-2014, 2016 | Ratio of annual number of live births to average population size in the same period. | Per thousand | The sample survey was conducted by a stratified multi-stage sampling method covering 1‰ population in China. |
|  |  | 2005, 2015 |  |  | The sample survey was conducted by a stratified multi-stage sampling method covering 1% population in China. |
|  |  | 2010 |  |  | Census data. |
| Female illiteracy | National Statistical Yearbooks | 2002-2004, 2006-2009, 2011-2014, 2016 | Ratio of women who were illiterate in the population aged 15 years or older. | Per hundred | The sample survey was conducted by a stratified multi-stage sampling method covering 1‰ population in China. |
|  |  | 2005, 2015 |  |  | The sample survey was conducted by a stratified multi-stage sampling method covering 1% population in China. |
|  |  | 2010 |  |  | Census data. |
| GRP per capita | National Statistical Yearbooks | 2002-2004, 2006-2009, 2011-2014, 2016 | Ratio of Gross Regional Product to population size. | Yuan per capita | The population size was obtained by a stratified multi-stage sampling survey covering 1‰ population in China. |
|  |  | 2005, 2015 |  |  | The population size was obtained by a stratified multi-stage sampling survey covering 1% population in China. |
|  |  | 2010 |  |  | The population size was obtained by census. |
| Urbanization rate | National Statistical Yearbooks | 2002-2004, 2006-2009, 2011-2014, 2016 | Ratio of urban population to general population. | Per hundred | The population size was obtained by a stratified multi-stage sampling survey covering 1‰ population in China. |
|  |  | 2005, 2015 |  |  | The population size was obtained by a stratified multi-stage sampling survey covering 1% population in China. |
|  |  | 2010 |  |  | The population size was obtained by census. |
| Child dependency ratio | National Statistical Yearbooks | 2002-2004, 2006-2009, 2011-2014, 2016 | Ratio of the number of children and adolescents to the number of the working-age population. | Per hundred | The population size was obtained by a stratified multi-stage sampling survey covering 1‰ population in China. |
|  |  | 2005, 2015 |  |  | The population size was obtained by a stratified multi-stage sampling survey covering 1% population in China. |
|  |  | 2010 |  |  | The population size was obtained by census |

GRP: gross reginal production; HE/GPBE: the ratio of health expenditure to general public budget expenditure

**Table S2 Provinces included in four regions in China**

| **Region** | **Province** |
| --- | --- |
| Eastern region | Beijing、Tianjin、Hebei、Shanghai、Jiangsu、Zhejiang、Fujian、Shandong、Guangdong、Hainan |
| Central region | Shanxi、Anhui、Jiangxi、Henan、Hubei、Hunan |
| Western region | Inner Mongolia、Guangxi、Chongqing、Sichuan、Guizhou、Yunnan、Tibet、Shaanxi、Gansu、Qinghai、Ningxia、Xinjiang |
| Northeast region | Liaoning、Jilin、Heilongjiang |

**Table S3** **Temporal trend of high-risk pregnancy rate based on Joinpoint regression model**

| **Country/**  **Province** | **Joinpoint 1** | | | **Joinpoint 2** | | | **Joinpoint 3** | | | **Entire time period** | |
| --- | --- | --- | --- | --- | --- | --- | --- | --- | --- | --- | --- |
|  | **Time period** | **APC (%, 95% CI)** | ***P*** | **Time period** | **APC (%, 95% CI)** | ***P*** | **Time period** | **APC (%, 95% CI)** | ***P*** | **(%, 95% CI)** | ***P*** |
| China | 1996-2002 | 8.19 (6.84, 11.30) | 0.0004 | 2002-2005 | 2.18 (0.57, 5.44) | 0.0164 | 2005-2016 | 5.98 (5.11,9.27) | 0.0044 | 6.06 (5.70, 6.51) | 0.0001 |
| Beijing | 2002-2009 | 3.80 (3.12, 5.52) | 0.0001 | 2009-2012 | -1.36 (-3.30, 1.44) | 0.3503 | 2012-2016 | 16.26 (14.02, 20.04) | 0.0001 | 6.05 (5.60, 6.54) | 0.0001 |
| Tianjin | 2002-2006 | -5.46 (-7.87, -2.94) | 0.0001 | 2006-2009 | 46.34 (42.11, 50.68) | 0.0001 | 2009-2016 | 7.23 (5.81, 8.54) | 0.0001 | 10.57 (9.93, 11.28) | 0.0001 |
| Hebei | 2002-2010 | 0.41 (-1.84, 1.39) | 0.5131 | 2010-2016 | 5.04 (3.43, 8.65) | 0.0001 | — | — | — | 2.37 (1.74, 2.98) | 0.0001 |
| Shanxi | 2002-2007 | 4.51 (1.31, 6.02) | 0.0124 | 2007-2010 | 14.70 (10.75, 16.88) | 0.0001 | 2010-2016 | 5.39 (3.76, 6.34) | 0.0008 | 7.00 (6.50, 7.44) | 0.0001 |
| Inner Mongolia | 2002-2006 | 4.44 (-0.76, 7.84) | 0.0840 | 2006-2009 | 10.44 (4.37, 13.31) | 0.0001 | 2009-2016 | 5.66 (0.41, 8.15) | 0.0440 | 6.32 (5.58, 7.00) | 0.0001 |
| Liaoning | 2002-2014 | 2.78 (0.28, 3.59) | 0.0436 | 2014-2016 | 9.32 (3.14, 13.00) | 0.0001 | — | — | — | 3.69 (2.85, 4.16) | 0.0001 |
| Jilin | 2002-2005 | -7.46 (-13.51, -2.69) | 0.0028 | 2005-2012 | 8.75 (6.81, 10.71) | 0.0001 | 2012-2016 | 23.32 (19.36, 29.40) | 0.0001 | 8.90 (7.80, 9.86) | 0.0001 |
| Heilong | 2002-2005 | -8.04 (-17.45, -2.06) | 0.0040 | 2005-2016 | 10.54 (9.44, 11.91) | 0.0001 | — | — | — | 6.27 (5.44, 7.30) | 0.0001 |
| Shanghai | 2002-2004 | -27.37 (-33.41, -16.19) | 0.0001 | 2004-2011 | 2.91 (-0.49, 7.16) | 0.0868 | 2011-2016 | 24.63 (19.30, 33.45) | 0.0001 | 4.84 (3.55, 6.51) | 0.0001 |
| Jiangsu | 2002-2007 | -0.86 (-9.71, 2.55) | 0.4071 | 2007-2010 | 12.39 (5.82, 17.25) | 0.0072 | 2010-2016 | 1.01 (-5.66, 3.07) | 0.6039 | 2.66 (1.41, 3.59) | 0.0001 |
| Zhejiang | 2002-2014 | 3.14 (1.73, 3.57) | 0.0068 | 2014-2016 | 8.83 (3.76, 11.39) | 0.0001 | — | — | — | 3.94 (3.31, 4.27) | 0.0001 |
| Anhui | 2002-2006 | -5.41 (-20.52, 2.91) | 0.1692 | 2006-2016 | 12.73 (10.33, 17.60) | 0.0001 | — | — | — | 7.22 (5.69, 9.26) | 0.0001 |
| Fujian | 2002-2016 | 6.24 (4.97, 7.48) | 0.0001 | — | — | — | — | — | — | 6.24 (4.97, 7.48) | 0.0001 |
| Jiangxi | 2002-2006 | -1.65 (-6.02, 0.48) | 0.1224 | 2006-2016 | 3.30 (2.71, 4.26) | 0.0001 | — | — | — | 1.86 (1.47, 2.35) | 0.0001 |
| Shandong | 2002-2009 | 4.67 (3.46, 6.50) | 0.0004 | 2009-2014 | -2.04 (-5.96, -0.23) | 0.0288 | 2014-2-16 | 13.26 (5.97, 18.40) | 0.0001 | 3.38 (2.68, 3.97) | 0.0001 |
| Henan | 2002-2006 | 3.37 (-3.37, 6.40) | 0.1628 | 2006-2009 | 22.37 (16.04, 26.08) | 0.0001 | 2009-2016 | 6.02 (4.30, 7.16) | 0.0004 | 8.54 (7.83, 9.25) | 0.0001 |
| Hubei | 2002-2016 | 2.82 (1.99, 3.64) | 0.0001 | — | — | — | — | — | — | 2.82 (1.99, 3.64) | 0.0001 |
| Hunan | 2002-2008 | 5.02 (3.90, 5.82) | 0.0001 | 2008-2016 | 8.79 (8.26, 9.48) | 0.0001 | — | — | — | 7.16 (6.89, 7.44) | 0.0001 |
| Guangdong | 2002-2014 | 4.52 (2.64, 5.17) | 0.0024 | 2014-2016 | 9.51 (4.81, 12.46) | 0.0001 | — | — | — | 5.22 (4.56, 5.60) | 0.0001 |
| Guangxi | 2002-2016 | 8.42 (7.54, 9.28) | 0.0001 | — | — | — | — | — | — | 8.42 (7.54, 9.28) | 0.0001 |
| Hainan | 2002-2006 | 0.88 (-9.22, 6.13) | 0.8294 | 2006-2016 | 9.65 (8.18, 13.81) | 0.0001 | — | — | — | 7.07 (5.74, 8.52) | 0.0001 |
| Chongqing | 2002-2016 | 6.54 (5.47, 7.59) | 0.0001 | — | — | — | — | — | — | 6.54 (5.47, 7.59) | 0.0001 |
| Sichuan | 2002-2008 | 10.72 (9.17, 12.63) | 0.0001 | 2008-2012 | 0.99 (-2.06, 3.40) | 0.5131 | 2012-2016 | 7.69 (5.53, 12.55) | 0.0001 | 6.99 (6.48, 7.58) | 0.0001 |
| Guizhou | 2002-2009 | 4.17 (2.46, 11.845) | 0.0032 | 2009-2016 | 0.16 (-6.73, 1.79) | 0.9890 | — | — | — | 2.15 (1.01, 3.28) | 0.0001 |
| Yunnan | 2002-2016 | 6.48 (3.81, 9.29) | 0.0001 | — | — | — | — | — | — | 6.48 (3.81, 9.29) | 0.0001 |
| Tibet | 2002-2008 | -7.14 (-21.05, -1.74) | 0.0092 | 2008-2016 | 3.35 (-0.30, 16.91) | 0.0708 | — | — | — | -1.28 (-3.30, 099) | 0.2563 |
| Shaanxi | 2002-2011 | 4.59 (4.14, 6.03) | 0.0001 | 2011-2016 | 2.62 (-0.52, 3.67) | 0.0756 | — | — | — | 3.88 (3.49, 4.30) | 0.0001 |
| Gansu | 2002-2005 | 7.93 (5.97, 10.56) | 0.0001 | 2005-2009 | 0.70 (-0.96, 2.15) | 0.3655 | 2009-2016 | 4.06 (3.50, 5.23) | 0.0001 | 3.90 (3.64, 4.23) | 0.0001 |
| Qinghai | 2002-2009 | -2.75 (-13.40, 0.94) | 0.1284 | 2009-2016 | 7.49 (3.53, 20.58) | 0.0028 | — | — | — | 2.25 (0.44, 4.04) | 0.0112 |
| Ningxia | 2002-2006 | 1.22 (-2.09, 2.65) | 0.2755 | 2006-2009 | 8.69 (6.14, 10.21) | 0.0001 | 2009-2016 | 3.52 (2.44, 4.08) | 0.0056 | 3.94 (3.58, 4.26) | 0.0001 |
| Xinjiang | 2002-2009 | 18.97 (14.94, 26.31) | 0.0001 | 2009-2016 | 3.85 (-1.94, 7.44) | 0.1288 | — | — | — | 11.15 (9.22, 13.10) | 0.0001 |

**Note:** HRPR: high-risk pregnancy rate. CI: confidence interval. APC: annual percent change. AAPC: average annual percent change

**Table S4 Predicted value of high-risk pregnancy rate in mainland China from 2017 to 2030**

| **Year** | **Predicted HRPR** | **95% CI** |
| --- | --- | --- |
| 2017 | 26.20% | (25.18%, 27.22%) |
| 2018 | 27.41% | (25.58%, 29.25%) |
| 2019 | 28.48% | (25.92%, 31.03%) |
| 2020 | 29.47% | (26.29%, 32.65%) |
| 2021 | 30.43% | (26.69%, 34.16%) |
| 2022 | 31.37% | (27.13%, 35.60%) |
| 2023 | 32.30% | (27.61%, 36.98%) |
| 2024 | 33.22% | (28.12%, 38.32%) |
| 2025 | 34.15% | (28.66%, 39.63%) |
| 2026 | 35.07% | (29.23%, 40.91%) |
| 2027 | 35.99% | (29.81%, 42.17%) |
| 2028 | 36.91% | (30.41%, 43.41%) |
| 2029 | 37.83% | (31.03%, 44.64%) |
| 2030 | 38.76% | (31.66%, 45.85%) |

**Note:** HRPR: high-risk pregnancy rate

**Table S5 Collinearity diagnostic of included independent variables**

| **Variables** | **Variance inflation factor** | |
| --- | --- | --- |
|  | **All variables** | **Excluding health staff per 1000 population and urbanization rate** |
| Hospital beds per 1000 population | 4.1690 | 2.1799 |
| Birth rate, ‰ | 3.2842 | 3.1346 |
| Female illiteracy, % | 1.9000 | 1.8516 |
| Log of GRP per capita | 6.6053 | 2.7624 |
| Urbanization rate, % | 7.6711 | − |
| HE/GPBE | 1.7395 | 1.7260 |
| Child dependency ratio | 4.9225 | 4.8974 |
| Health staff per 1000 population | 5.5658 | − |

**Note:** GRP: gross reginal production; HE/GPBE: the ratio of health expenditure to general public budget expenditure.


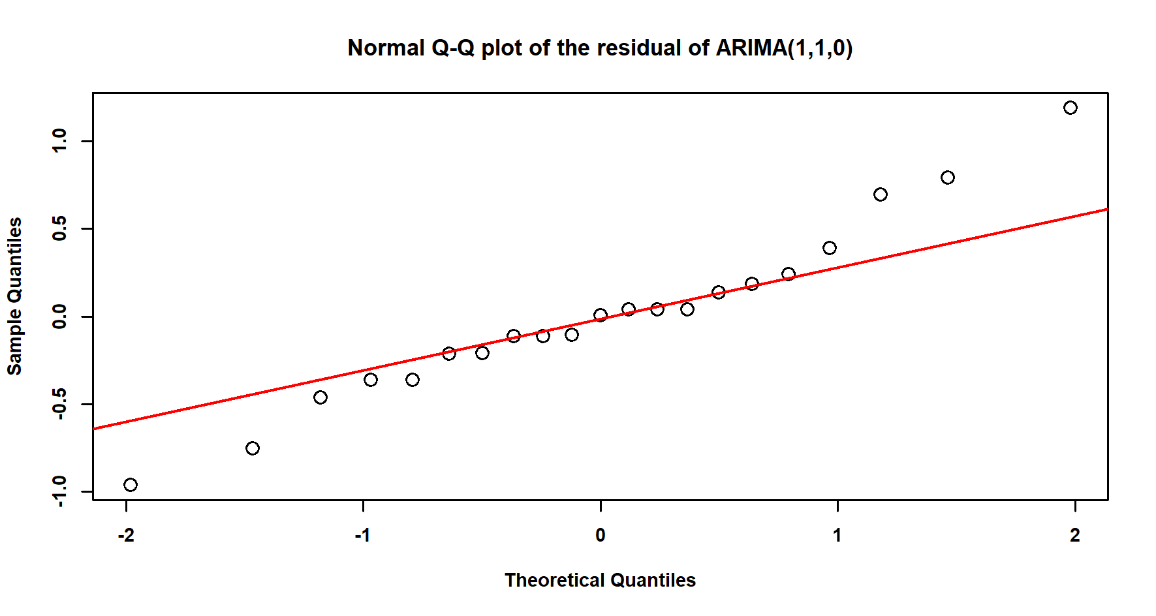


**Figure S1 Normal Q-Q plot for the residual of ARIMA (1,1,0)**
